# Supplementary material for: Topical application of calcitonin gene-related peptide as a regenerative, antifibrotic, and immunomodulatory therapy for corneal injury
Source: Commun Biol. 2024 Mar 4;7:264. doi: 10.1038/s42003-024-05934-y (PMC10912681; doi:10.1038/s42003-024-05934-y)
Supplement: Supplementary file 2 — Supplementary Information [file 42003_2024_5934_MOESM2_ESM.pdf]

Title: Topical application of calcitonin gene-related peptide as a regenerative, antifibrotic, and immunomodulatory therapy for corneal injury

Authors: Asmaa A. Zidan[1], Shuyan Zhu[1], Elsayed Elbasiony[1], Sheyda Najafi[1], Zhirong Lin[1], Rohan Bir Singh[1], Amirreza Naderi[1], Jia Yin[1*].

Affiliation: 1- Schepens Eye Research Institute of Massachusetts Eye and Ear, Department of Ophthalmology, Harvard Medical School, Boston, MA, 02114

***Corresponding Author**

Jia Yin, MD, PhD, MPH, Schepens Eye Research Institute of Massachusetts Eye and Ear, Department of Ophthalmology, Harvard Medical School, 20 Staniford St., Boston MA 02114.

Email: jia_yin@meei.harvard.edu ; Phone no: +1-617-912-0272

Keywords: Corneal wound healing; corneal injury; corneal opacity; ocular trauma; CGRP, calcitonin gene-related peptide

Supplementary Figure 1. CGRP preserved normal corneal endothelial cell (CEnC) morphology.

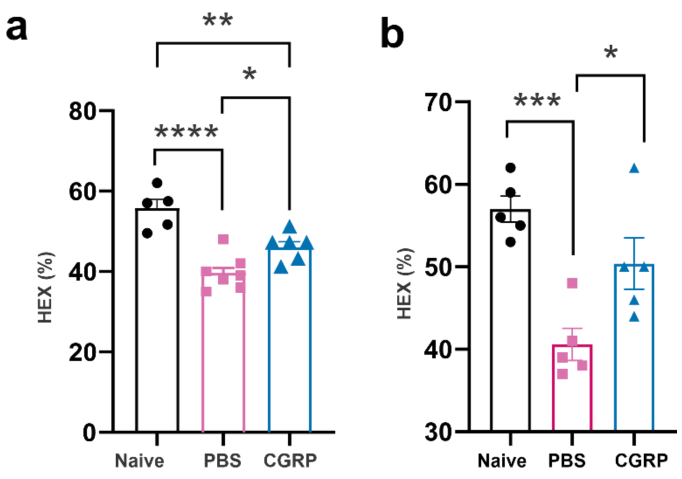

a CEnC pleomorphism, or the percentage of normal hexagonal shaped cells over total cells (HEX %), was analyzed using in vivo confocal microscopy images of corneal endothelial cells. While pleomorphism in the injured PBS treated corneas was increased, CGRP treatment preserved the normal morphology. **b** CEnC pleomorphism was analyzed using images of corneal endothelium whole mount stained by ZO-1. Results were similar to Figure A. N=5 mice per group. The data are presented as mean ± standard error of mean and was determined by one-way ANOVA test with pairwise comparison. $*p<0.05$, $***p<0.001$, $****p<0.0001$.

Supplementary Figure 2. Short term application of CGRP alleviates corneal opacity but does not impact thickness.

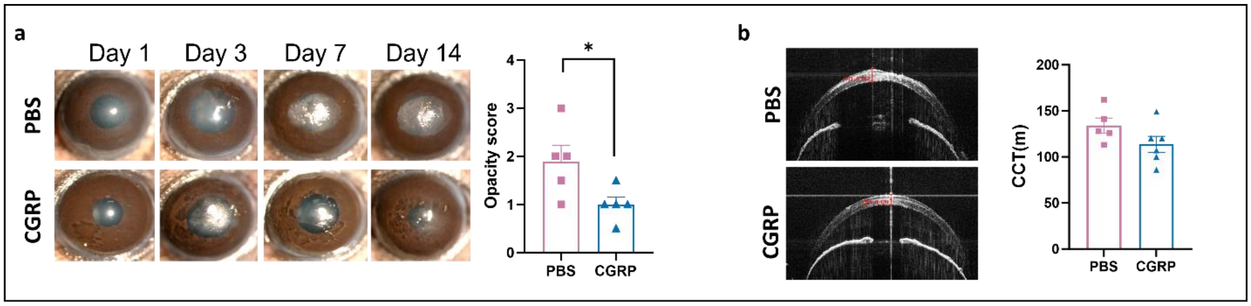

a Representative slit lamp photographs of PBS and CGRP-treated eyes up to 5 days after injury. The corneas of the PBS-treated controls showed progressive stromal opacification, whereas CGRP treatment showed significantly lower corneal opacity. **b**. Representative AS-OCT images and analysis showed significant increase in central corneal thickness (CCT) and stromal hyperreflectivity in PBS-treated mice, whereas they were comparable to CGRP treated group on days 14 post-injury. (n=5 per group). The data are represented as mean±SEM. The statistical significance was determined by unpaired t-test, * $p<0.05$.

Supplementary Figure 3. Expression of CGRP receptors by corneal cells.

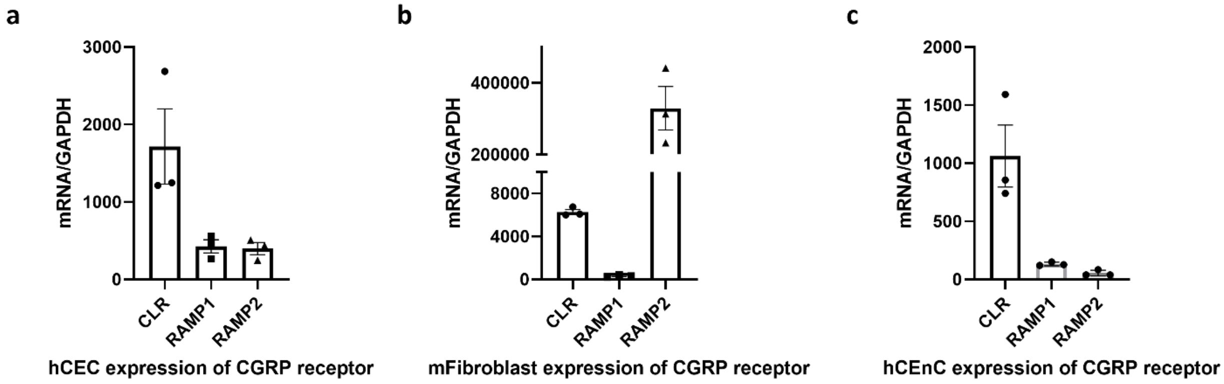

The RT-PCR analysis shows that corneal epithelial cells (hCEC, **a**), corneal fibroblast (**b**) and corneal endothelial cells **(c)** express the CGRP receptors components CLR, RAMP1 and RAMP2.

Supplementary Figure 4. Analysis of western blot on TGF-β1 expression in vivo and αSMA expression in vivo and in vitro.

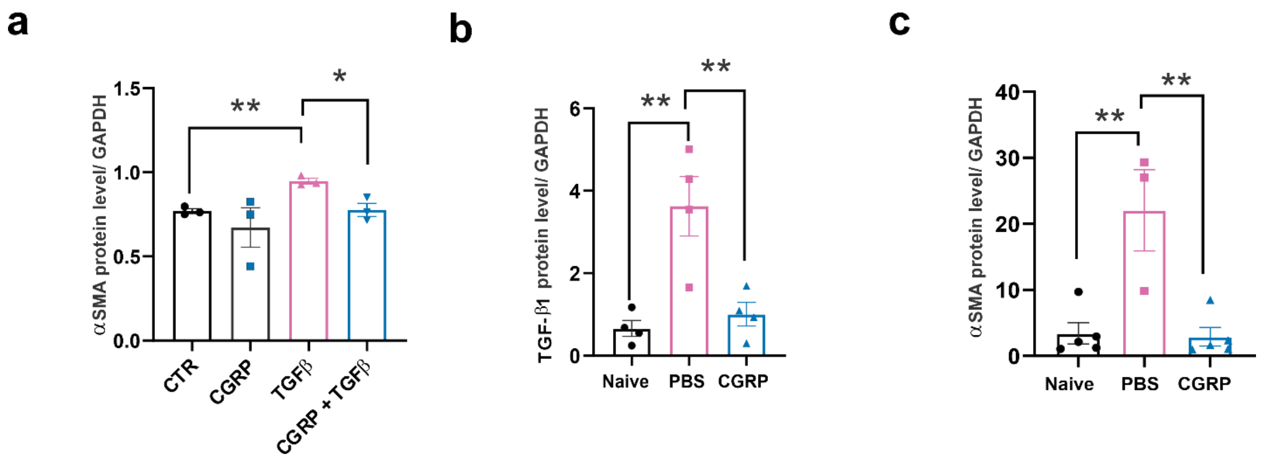

a CGRP significantly suppressed TGF-β1-induced αSMA expression in vitro. **b** CGRP significantly decreased TGF-β1 expression in corneal tissues on day 5 after injury compared to the PBS control. **C.** CGRP significantly decreased αSMA expression in corneal tissues on day 5 after injury compared to the PBS control. n=4 per group. The data are presented as mean ± SEM and was determined by one-way ANOVA test with pairwise comparison. *$p<0.05$, **$p<0.01$.

Supplementary Figure 5. CGRP topical application failed to increase the CGRP concentration in the Aqueous humor.

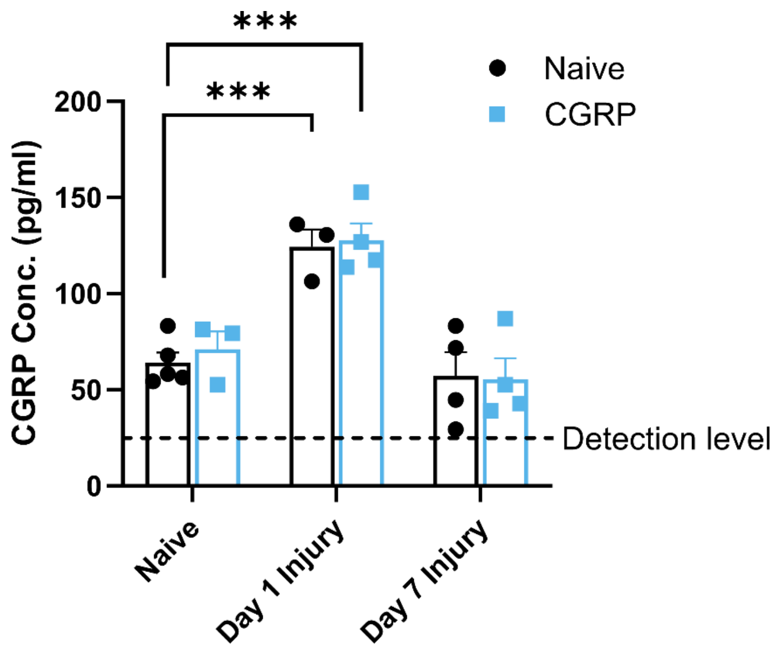

Aqueous humor was collected from naïve, one day and seven days post-injury mice and CGRP concentration was determined using CGRP ELISA Kit. Surprisingly, we noticed a significant increase in CGRP conc. On day 1 post-injury compared to naïve mice. By day 7- post injury, the CGRP concentration was back to normal. Then, CGRP was applied topically for 30 minutes before aqueous humor collection in either naïve mice, or injured mice on day 1 or day 7. No significant difference in the AH concentration was noticed in response to the CGRP application. (Two eyes were pooled together to generate enough volume/sample, n=4/group). The data are represented as mean±SEM. The statistical significance was determined by unpaired t-test, $p<0.001$.

Supplementary Figure 6. CGRP topical application suppresses ocular hyperalgesia (eye-wiping test) on day 3 post-injury.

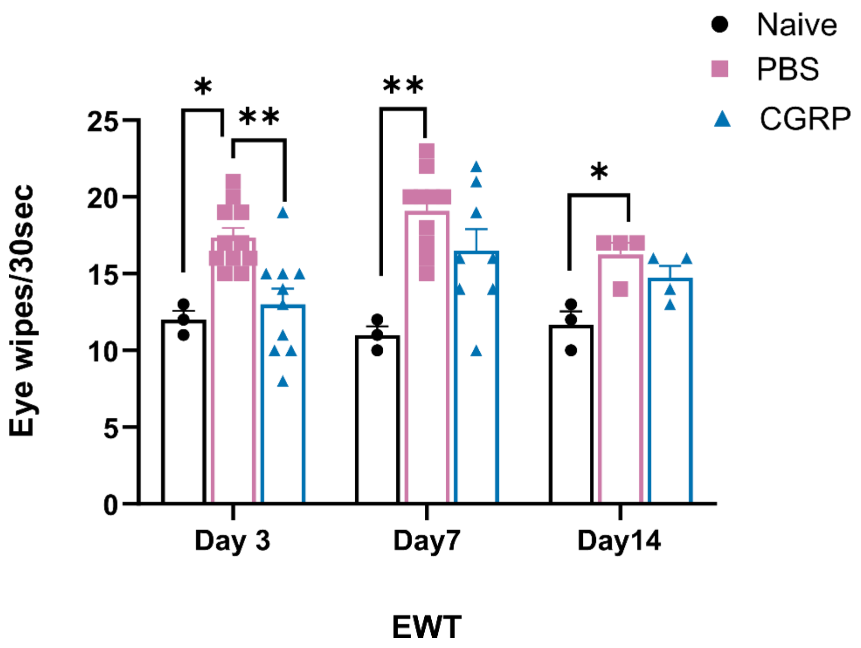

Ocular hyperalgesia was assessed using the eye-wiping test [1,2]. Briefly, a single drop of 2M NaCl was instilled into the animal's eye, and the count of eye wipes with the ipsilateral forelimb was recorded over a 30-second period.

Mechanical injury induced increased eye wipes number up to day 14. CGRP topical administration significantly suppressed the eye wipes on day 3 post-injury. The data are presented as mean ±SEM and was determined by one-way ANOVA test with pairwise comparison, *p<0.05, **p<0.01.

Supplementary Figure 7. The flow cytometry gating strategy.

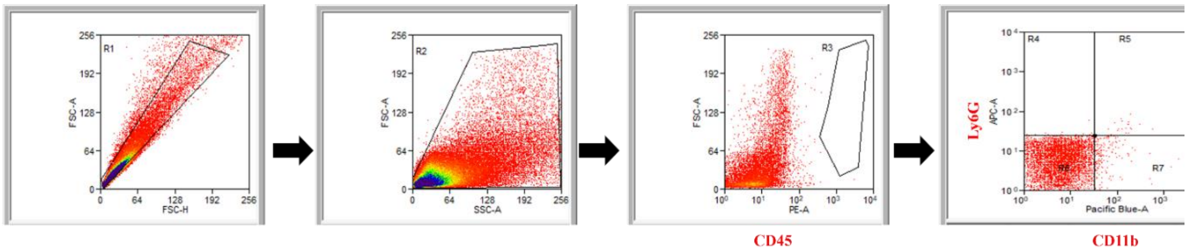

The figure illustrates the gating strategy utilized for identifying inflammatory cells (CD45+) within the cornea, as well as distinguishing the subpopulations of neutrophils (CD11b+Ly6G+) and macrophages (CD11b+Ly6G-).

Supplementary Figure 8. Uncropped blot images.

Fig. 4g

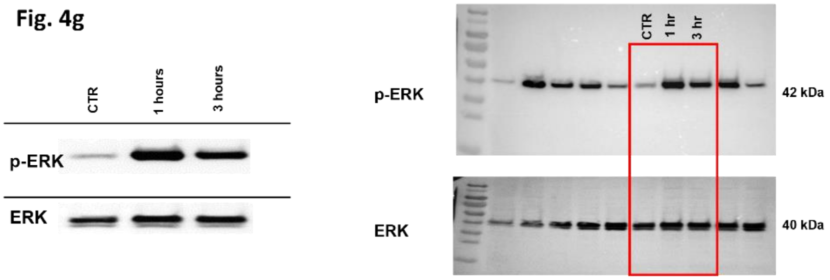

Fig. 5b

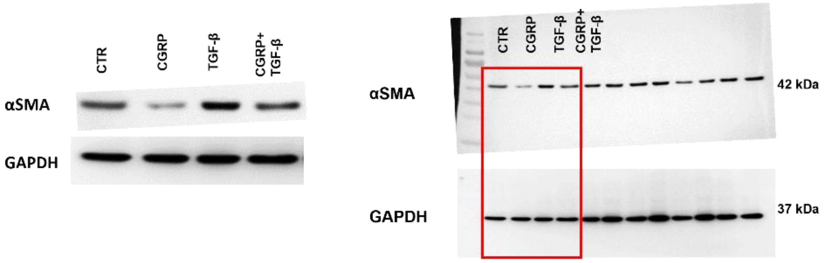

Fig. 5e

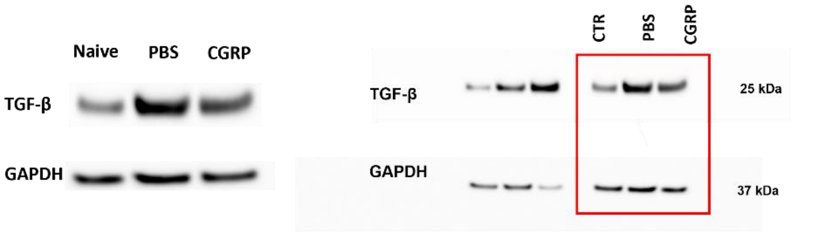

Fig. 5h

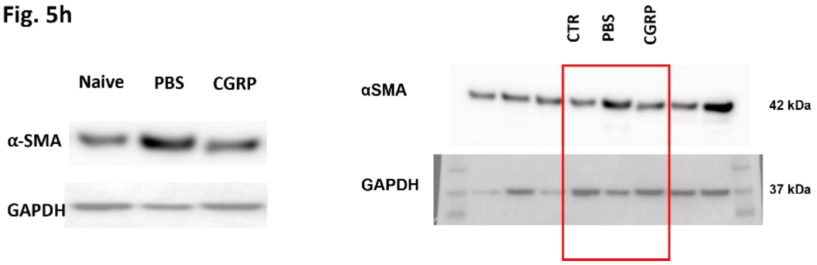

Supplementary Table 1: The primers used in the RT-qPCR analysis.

**Name**	**Gene name**	**Primer**
GAPDH	*GAPDH*	Mm99999915_g1
CLR	*Calcr*	Mm00432282_m1
RAMP1	*RAMP1*	Mm00489796_m1
RAMP2	*RAMP2*	Mm00490256_g1
Laminin	*LAMA3*	Hs00165042_m1
Alpha SMA	*Acta-2*	Mm00725412_s1
TGF-beta1	*Tgfb1*	Mm01178820_m1
Na/K ATPase α1	*ATP1A1*	Mm00523255_m1
Na/K ATPase α3	*ATP1A3*	Mm00523430_m1
Caspase 3 (human)	*CAS3*	Hs00234387_m1
BAX (human)	*BAX*	Hs00180269_m1
GAPDH (human)	*GAPDH*	Hs02786624_g1
CXCL1	*CXCL1*	Mm00434772_m1
TNFα1	*TNFA*	Mm00443258_m1
MMP9	*MMP9*	Mm00442991_m1
IL-1beta	*Il1b*	Mm00434228_m1

All the listed Taqman primers were purchased from Thermo-Fischer Scientific Company.

Supplementary Table 2: The antibodies used in the immunohistochemistry (IHC) and western blot (WB).

Antibody	Manufacturer	Cat#
**Primary Antibodies (IHC/WB)**		
Ki-67 Monoclonal Antibody (SolA15), FITC,	Invitrogen	11-5698-82
p-ERK Antibody	Thermofisher	14-9109-82
ERK1/ERK2 Antibody	Thermofisher	82380
Laminin Antibody	Thermofisher	PA1-16730
Alpha-Smooth Muscle Actin Antibody	Thermofisher	14-9760-82
TGF beta-1 Monoclonal Antibody	Thermofisher	MA1-21595
Propidium iodide	Sigma-Aldrich	P4864
Annexin V-FITC	Biolegend	640906
Sodium Potassium ATPase Recombinant Rabbit Monoclonal Antibody (ST0533	Thermofisher	MA5-32184
Zonula Occludens Antibody	Thermofisher	33-9100
**Secondary Antibodies (IHC)**		
Anti-Mouse IgG1 Nano Recombinant Secondary Antibody, Alexa Fluor™ 488	Thermofisher	SA5-10329
Goat anti-Rabbit IgG Fc, Cross-Adsorbed Secondary Antibody, Alexa Fluor™ 488	Thermofisher	A78953
Goat anti-Rabbit IgG (H+L) Highly Cross-Adsorbed Secondary Antibody, Alexa Fluor™ Plus 594	Thermofisher	A32740
Goat anti-Mouse IgG (H+L) Cross-Adsorbed Secondary Antibody, Alexa Fluor™ 594	Thermofisher	A-11005
**Secondary Antibodies (WB)**		
Goat anti-Rabbit IgG (H+L) secondary antibody, HRP	Thermofisher	32460
Goat anti-Mouse IgG (H+L) Secondary Antibody, HRP	Thermofisher	31430
GADPH loading control antibody-HRP	Thermofisher	MA5-15738-HRP

Supplementary References:

1 Farazifard, R., Safarpour, F., Sheibani, V. & Javan, M. Eye-wiping test: a sensitive animal model for acute trigeminal pain studies. *Brain Res Brain Res Protoc* **16**, 44-49 (2005). https://doi.org:10.1016/j.brainresprot.2005.10.003

2 Nazeri, M., Zarei, M. R., Pourzare, A. R., Ghahreh-Chahi, H. R., Abareghi, F. & Shabani, M. Evidence of Altered Trigeminal Nociception in an Animal Model of Fibromyalgia. *Pain Med* **19**, 328-335 (2018). https://doi.org:10.1093/pm/pnx114
