# Supplementary material for: Topical application of calcitonin gene-related peptide as a regenerative, antifibrotic, and immunomodulatory therapy for corneal injury
Source: Commun Biol. 2024 Mar 4;7:264. doi: 10.1038/s42003-024-05934-y (PMC10912681; doi:10.1038/s42003-024-05934-y)
Supplement: Supplementary file 5 — Reporting Summary [file 42003_2024_5934_MOESM5_ESM.pdf]

nature portfolio

Corresponding author(s): Jia Yin

Last updated by author(s): Feb 5, 2024

Reporting Summary

Nature Portfolio wishes to improve the reproducibility of the work that we publish. This form provides structure for consistency and transparency in reporting. For further information on Nature Portfolio policies, see our Editorial Policies and the Editorial Policy Checklist.

Statistics

For all statistical analyses, confirm that the following items are present in the figure legend, table legend, main text, or Methods section.

n/a	Confirmed	
☐	☒	The exact sample size (*n*) for each experimental group/condition, given as a discrete number and unit of measurement
☐	☒	A statement on whether measurements were taken from distinct samples or whether the same sample was measured repeatedly
☐	☒	The statistical test(s) used AND whether they are one- or two-sided<br>*Only common tests should be described solely by name; describe more complex techniques in the Methods section.*
☒	☐	A description of all covariates tested
☐	☒	A description of any assumptions or corrections, such as tests of normality and adjustment for multiple comparisons
☐	☒	A full description of the statistical parameters including central tendency (e.g. means) or other basic estimates (e.g. regression coefficient) AND variation (e.g. standard deviation) or associated estimates of uncertainty (e.g. confidence intervals)
☐	☒	For null hypothesis testing, the test statistic (e.g. *F*, *t*, *r*) with confidence intervals, effect sizes, degrees of freedom and *P* value noted<br>*Give P values as exact values whenever suitable.*
☒	☐	For Bayesian analysis, information on the choice of priors and Markov chain Monte Carlo settings
☒	☐	For hierarchical and complex designs, identification of the appropriate level for tests and full reporting of outcomes
☒	☐	Estimates of effect sizes (e.g. Cohen's *d*, Pearson's *r*), indicating how they were calculated

Our web collection on statistics for biologists contains articles on many of the points above.

Software and code

Policy information about availability of computer code

Data collection	CD-15/CellChekD+ (Konan Medical) was used to collect the corneal endothelial cells' morphology parameters.
Data analysis	Data analysis was done using FlowJO_v10, GraphPad Prism software 9.4.1 (458) and ImageJ software Version 1.52.

For manuscripts utilizing custom algorithms or software that are central to the research but not yet described in published literature, software must be made available to editors and reviewers. We strongly encourage code deposition in a community repository (e.g. GitHub). See the Nature Portfolio guidelines for submitting code & software for further information.

Data

Policy information about availability of data

All manuscripts must include a data availability statement. This statement should provide the following information, where applicable:

- Accession codes, unique identifiers, or web links for publicly available datasets
- A description of any restrictions on data availability
- For clinical datasets or third party data, please ensure that the statement adheres to our policy

The source data behind the graphs in the manuscript were shown in Supplementary Data 1. The other datasets generated and/or analyzed during the current study are available upon request from the corresponding author.

Research involving human participants, their data, or biological material

Policy information about studies with human participants or human data. See also policy information about sex, gender (identity/presentation), and sexual orientation and race, ethnicity and racism.

Reporting on sex and gender	N/A
Reporting on race, ethnicity, or other socially relevant groupings	N/A
Population characteristics	N/A
Recruitment	N/A
Ethics oversight	N/A

Note that full information on the approval of the study protocol must also be provided in the manuscript.

Field-specific reporting

Please select the one below that is the best fit for your research. If you are not sure, read the appropriate sections before making your selection.

☒ Life sciences ☐ Behavioural & social sciences ☐ Ecological, evolutionary & environmental sciences

For a reference copy of the document with all sections, see nature.com/documents/nr-reporting-summary-flat.pdf

Life sciences study design

All studies must disclose on these points even when the disclosure is negative.

Sample size	Based on our preliminary of opacity score, the mean values of control and CGRP groups are 3.1 and 1.9 respectively, the standard deviation is 0.9. A two-sided P value of 0.05 and a power of 0.8 will require 5 mice per group. For in vitro testing, a minimum sample size of 3 in each group was determined to adequately perform statistical analysis.
Data exclusions	No data points were excluded.
Replication	All experiments were repeated at least twice and all attempts at replication were successful.
Randomization	Corneal injury was induced simultaneously, followed by random assignment to either the PBS group or the CGRP treatment group.
Blinding	Clinical assessment of animal experiments was performed by a masked observer.

Reporting for specific materials, systems and methods

We require information from authors about some types of materials, experimental systems and methods used in many studies. Here, indicate whether each material, system or method listed is relevant to your study. If you are not sure if a list item applies to your research, read the appropriate section before selecting a response.

Materials & experimental systems

n/a	Involved in the study	
☐	☒	Antibodies
☐	☒	Eukaryotic cell lines
☒	☐	Palaeontology and archaeology
☐	☒	Animals and other organisms
☒	☐	Clinical data
☒	☐	Dual use research of concern
☒	☐	Plants

Methods

n/a	Involved in the study	
☒	☐	ChIP-seq
☐	☒	Flow cytometry
☒	☐	MRI-based neuroimaging

Antibodies

Antibodies used	CD45 antibody : Biolegend, catalog# 103101, Clone 30-F11<br>PE anti-mouse Ly-6G , Biolegend, catalog#127607

PerCP/Cyanine5.5 anti-mouse/human CD11b Antibody, Biolegend, catalog# 101227
APC/Cy7-conjugated anti-CD45 antibody: BioLegend, catalog# 103116, Clone 30-F11
APC/Cyanine7 Rat IgG2b, κ Isotype Ctrl Antibody: Biolegend, catalog#400624, Clone RTK4530
Ki-67 Monoclonal Antibody (SolA15), FITC conjugated, Invitrogen, catalog#11-5698-82
p-ERK Antibody, Thermofisher, catalog#14-9109-82
ERK1/ERK2 Antibody ,Thermofisher,catalog# 82380
Laminin Antibody, Thermofisher, catalog#PA1-16730
Alpha-Smooth Muscle Actin Antibody, Thermofisher,catalog#14-9760-82
TGF beta-1 Monoclonal Antibody, ThermoFischer catalog#MA1-21595
Sodium Potassium ATPase Recombinant Rabbit Monoclonal Antibody, Thermofisher, catalog#MA5-32184
Zonula Occludens Antibody, Thermofisher,catalog#33-9100

Validation

Alpha-SMA antibody: Thermofisher, catalog#14-9760-82, clone 1A4. Monoclonal antibody recognizes muscle actin; specifically the alpha and gamma form in human, mouse, and rat. It has been tested in immunohistochemistry by the manufacturer and used in published studies. Host/Isotype - Mouse / IgG2a, kappa.

CD45 antibody: Biolegend, catalog# 103101, Clone 30-F11. Monoclonal antibody recognizes mouse CD45 and has been verified in immunohistochemistry by the manufacturer. Host/Isotype - Rat IgG2b, κ

APC/Cy7-conjugated anti-CD45 antibody: BioLegend, catalog# 103116, Clone 30-F11. Monoclonal antibody recognizes mouse CD45. It has been quality-tested in flow cytometry by the manufacturer. Host/Isotype - Rat IgG2b, κ

PE Monoclonal antibody anti-mouse Ly-6G , Biolegend, catalog#127607, clone 1A8. It has been quality-tested in flow cytometry by the manufacturer. Host/Isotype -Rat IgG2a, κ

PerCP/Cyanine5.5 anti-mouse/human CD11b Antibody, Biolegend, catalog# 101227, clone M1/70. It has been quality-tested in flow cytometry by the manufacturer. Host/Isotype -Rat IgG2b, κ

APC/Cyanine7 Rat IgG2b, κ Isotype Ctrl Antibody: Biolegend, catalog#400624, Clone RTK4530. Monoclonal antibody was chosen as an isotype control after screening on a variety of resting, activated, live, and fixed mouse, rat and human tissues and has been quality-tested in flow cytometry by the manufacturer. Host/Isotype - Rat IgG2b, κ.

FITC conjugated Ki-67 Monoclonal Antibody: Invitrogen, catalog#11-5698-82, clone SolA15. This has been verified in immunohistochemistry by the manufacturer. Host/isotype - Rat / IgG2a, kappa.

p-ERK Antibody: Thermofisher, catalog#14-9109-82, clone MILAN8R.Monoclonal antibody recognizes p-ERK and has been verified in immunohistochemistry and western blot by the manufacturer. Host/Isotype - R Mouse / IgG1, kappa

ERK1/ERK2 Antibody: Thermofisher,catalog# 82380. Polyclonal antibody recocgizes ERK1 and ERK2 (involved in the regulation of meiosis, mitosis). This has been verified in immunohistochemistry and western blot by the manufacturer. Host/Isotype - Rabbit / IgG.

Laminin Antibody, Thermofisher, catalog#PA1-16730. This antibody is pan-specific and reacts well with all Laminin isoforms tested: Laminin-1 (alpha-1, beta-1, and gamma-1) and Laminin-2 (alpha-2, beta-1, and gamma-1). This has been verified in immunohistochemistry by the manufacturer. Host/Isotype - Rabbit / IgG.

Recombinant Monoclonal TGF-beta Antibody,Thermofisher,MA1-21595, clone TB21. This has been verified in western blot and immunohistochemistry by the manufacturer and publications. Host/Isotype - Mouse IgG1.

Sodium Potassium ATPase Recombinant Rabbit Monoclonal Antibody, Thermofisher, catalog#MA5-32184, clone ST0533.This has been verified in immunohistochemistry by the manufacturer. Host/Isotype -Rabbit / IgG

Zonula Occludens Monoclonal Antibody, Thermofisher,catalog#33-9100, clone ZO1-1A12. This has been verified in immunohistochemistry by the manufacturer. Host/Isotype -Mouse / IgG1.

Eukaryotic cell lines

Policy information about cell lines and Sex and Gender in Research

Cell line source(s)

Mice corneal fibroblast cell line MK/T-1 was kindly provided by Dr. Sunil K. Chauhan of the Schepens Eye Research Institute of Mass Eye and Ear. The cell line was derived from mice corneal stromal cells by transfection with a human telomerase reverse transcriptase (hTERT).

Human corneal endothelial cell line hCEnC-21T was kindly provided by Dr. Ula Jurkunas of the Schepens Eye Research Institute of Mass Eye and Ear. The cell line was derived from a 21-year-old male donor and transduction with telomerase yielded this highly proliferative cell line.

Telomerase-immortalized human corneal epithelium cells (hCEC) were kindly provided by Dr. Pablo Argueso of Tufts Medical Center. The cell line was derived from human corneoscleral donor rims, provided by Roger Steinert and Ann Bajart of Ophthalmic Consultants of Boston.The cells were immortalized by abrogation of p16 control and p53 function before immortalization by expression of hTERT.

Authentication

Human corneal endothelial cell line hCEnC-21T: In the original publication by Dr. Jurkunas (PMID: 23284695), the cell line was authenticated to be devoid of oncogenic transformation and retain critical corneal endothelial cell characteristics and functionality. The cell line has since been used in multiple studies and reported in the literature.

	Telomerase-immortalized human corneal epithelium cells (hCEC): In the original publication by Dr. Argueso and Dr Russo (PMID: 12766048), the corneal cell line expressed keratins K3 and K12, the keratins that are corneal-epithelial-specific, and K19. The cell line has since been used in multiple studies and reported in the literature.<br><br>Mice corneal fibroblast cell line MK/T-1: In the original publication (PMID: 11344338), the MK/T-1 cells express vimentin, tubulin, lumican, mimecan, decorin and collagen I, but not keratocan. Exposure of the MK/T-1 cells to TGF-b induces the expression of smooth muscle a-actin (ASMA), the activation of MAP Kinase (p38-MAPK) and morphological changes consistent with cytoskeletal reorganization.
Mycoplasma contamination	The cell lines were not tested for mycoplasma contamination.
Commonly misidentified lines<br>(See ICLAC register)	N/A

Animals and other research organisms

Policy information about studies involving animals; ARRIVE guidelines recommended for reporting animal research, and Sex and Gender in Research

Laboratory animals	C57BL/6 mice, male and females, age 6-8 weeks, were obtained from the Charles River Laboratories. The mice were kept at a 10/14-hour dark/light cycle with ambient temperature 21-23°C, and humidity 40-60%.
Wild animals	The study did not involve wild animals.
Reporting on sex	In the current study, adult male and females mice between the ages of 6 and 8 weeks were used.
Field-collected samples	The study did not involve samples collected from the field.
Ethics oversight	Animal Care Committee of the Schepens Eye Research Institute

Note that full information on the approval of the study protocol must also be provided in the manuscript.

Flow Cytometry

Plots

Confirm that:

☒ The axis labels state the marker and fluorochrome used (e.g. CD4-FITC).

☒ The axis scales are clearly visible. Include numbers along axes only for bottom left plot of group (a 'group' is an analysis of identical markers).

☒ All plots are contour plots with outliers or pseudocolor plots.

☒ A numerical value for number of cells or percentage (with statistics) is provided.

Methodology

Sample preparation	Single cell suspension from the harvested corneas was prepared by incubating the corneas in collagenase type IV (Sigma-Aldrich Inc., St. Louis, MO) and DNase I (Roche Corp., Basel, Switzerland) for 45 minutes at 37°C. Subsequently, the cells were passed through a 70-µm cell strainer (Corning Inc., Corning, NY). Then the cells were stained following the manufacturer recommendation for each antibody . The appropriate isotypes were utilized as controls for the antibodies.
Instrument	LSRII flow cytometer (BD Biosciences, San Jose, CA)
Software	FCS Express software (De Novo Software, Los Angeles, CA).
Cell population abundance	Cell population and purity were determined post-sort with negative controls.
Gating strategy	Gating strategies were confirmed in preliminary experiments.

☒ Tick this box to confirm that a figure exemplifying the gating strategy is provided in the Supplementary Information.
